# Supplementary material for: Population dynamics of sugarcane borers, Diatraea spp., under different climatic scenarios in Colombia
Source: PLoS One. 2021 Jan 15;16(1):e0244694. doi: 10.1371/journal.pone.0244694 (PMC7810321; doi:10.1371/journal.pone.0244694)
Supplement: Attachment — Submitted filename: Comments for Authors.docx [file pone.0244694.s001.docx]

**Comments for Authors**

The manuscript “population dynamics of Diatraea spp. under different climate offer
conditions in Colombia” present an interesting study on how climate change is expected to affect the distribution of sugar cane stem borers, Diatraea spp. The manuscript is well structured and nicely presented. However, I have some observations and comments for the authors as follows.

Abstract

Authors should mention for which scenarios the study was conducted for.

Authors should include more key findings from the study in the abstract.

Introduction

1. The Earth's temperature increased between 0. 7°C from 1900, 1. 3°C from 1950 and 1. 8°C
   over the past 35 years. This sentence should be rephrased for better understanding for readers.
2. Many of the sentences in this section are too long; authors should make them shorter or split them.

**Materials and Methods**

1. Change ‘The climate change model’ to ‘The global climate model (GCM)’
2. Why was this BCC_CSM1 chosen?
3. Change (CPR) to (RCP)
4. Change ‘depending on the number’ to ‘depending on the amount’
5. Remove ‘it’ in the sentence: MODESTR®, like MAXENT®, (Elith *et al*., 2011, Phillips and Dudik, 2008) it
6. Authors should split the paragraph that started with: To generate the distribution maps and to see the effect for each variable in the distribution of *Diatraea* spp., MODESTR® (http://www.ipezes/ModestR)..........

**Results**

1. Why are legends not given in the Figures in the results?
2. Authors should label the axis of Figure 5.
3. Label axis of Figure 6.
4. The authors mentioned anthropogenic factors affecting the *Diatraea* spp. Under the section: Factors affecting *Diatraea* spp. Richness. Are there other anthropogenic factors aside from the primary production (PP) and vegetation index e.g. does factors such as pest control affect them?
5. Under section distribution maps: Change this word ‘affection’ to ‘impaction’
6. From section 3.4 authors should check the figure numberings and correct them.

**Other comments**

Authors should include lines numbering in manuscript the next times

There are grammatical and typographical errors in the manuscript. Authors should check and correct for these.
