# Supplementary material for: Population dynamics of sugarcane borers, Diatraea spp., under different climatic scenarios in Colombia
Source: PLoS One. 2021 Jan 15;16(1):e0244694. doi: 10.1371/journal.pone.0244694 (PMC7810321; doi:10.1371/journal.pone.0244694)
Supplement: Attachment — Submitted filename: Response to Reviewers.docx [file pone.0244694.s003.docx]

**PlosOne**

**Response to Reviewers**

**Journal Requirements**

1. Please ensure that your manuscript meets PLOS ONE's style requirements, including those for file naming.

PLOS ONES´s style requirements have been included

2. We suggest you thoroughly copyedit your manuscript for language usage, spelling, and grammar. If you do not know anyone who can help you do this, you may wish to consider employing a professional scientific editing service.

We hired Alexandra Walter, a professional Spanish-English translator with 30 years of experience with scientific publications at the International Center for Tropical Agriculture in Colombia - CIAT. Alexandra retired from CIAT in 2008 and works as a freelance editor.

3. We note that Figures in your submission contain map images which may be copyrighted. All PLOS content is published under the Creative Commons Attribution License (CC BY 4.0), which means that the manuscript, images, and Supporting Information files will be freely available online, and any third party is permitted to access, download, copy, distribute, and use these materials in any way, even commercially, with proper attribution. For these reasons, we cannot publish previously copyrighted maps or satellite images created using proprietary data, such as Google software (Google Maps, Street View, and Earth). For more information, see our copyright guidelines: <http://journals.plos.org/plosone/s/licenses-and-copyright>.

We used ModestR to design the maps, so they are original and not previously copyrighted. Fig 1 is a free available figure from the Cenicaña´s webpage whose source is accordingly cited.

4. Please provide a direct link to the source of the replacement map in Figure 1.

The distribution map of the weather stations was generated by QGIS 3.6.1, according to coordinates of the different stations. As the map was made using free software, there is no direct link to share.

5. We note that your Financial Disclosure statement is currently as follows: "Unfunded studies".

The Project was funded through resources from by the Colombian Institute for the Development of Science and Technology (COLCIENCIAS) by grant program number 647, in addition to support through the use of infrastructure and laboratories of the Colombian Sugarcane Research Center (*Centro de Investigación de la Caña de Azúcar de Colombia, Cenicaña*)

**Comments for Authors**

The manuscript “population dynamics of *Diatraea* spp. under different climate offer
conditions in Colombia” present an interesting study on how climate change is expected to affect the distribution of sugar cane stem borers, *Diatraea* spp. The manuscript is well structured and nicely presented. However, I have some observations and comments for the authors as follows.

Abstract

Authors should mention for which scenarios the study was conducted for.

Done

Authors should include more key findings from the study in the abstract.

More key findings have been included in the abstract

Introduction

1. The Earth's temperature increased between 0. 7°C from 1900, 1. 3°C from 1950 and 1. 8°C
   over the past 35 years. This sentence should be rephrased for better understanding for readers.

Sentence has been rephrased for better understanding

2. Many of the sentences in this section are too long; authors should make them shorter or split them.

Sentences have been edited and organized for better understanding

**Materials and Methods**

1. Change ‘The climate change model’ to ‘The global climate model (GCM)’

Done

2. Why was this BCC_CSM1 chosen?

Lines 138 – 141: “… according to the Colombian Institute of Hydrology, Meteorology and Environmental Studies (*Instituto de Hidrología, Meteorología y Estudios Ambientales*, IDEAM) this model fits well Colombian meteorological conditions.”

3. Change (CPR) to (RCP)

Done

4. Change ‘depending on the number’ to ‘depending on the amount’

Done

5. Remove ‘it’ in the sentence: MODESTR®, like MAXENT®, (Elith *et al*., 2011, Phillips and Dudik, 2008) it

Done

6. Authors should split the paragraph that started with: To generate the distribution maps and to see the effect for each variable in the distribution of *Diatraea* spp., MODESTR® (<http://www.ipezes/ModestR)>..........

Done

**Results**

1. Why are legends not given in the Figures in the results?

Legends have been added to figs

2. Authors should label the axis of Figure 5.

Done

3. Label axis of Figure 6.

Done

4. The authors mentioned anthropogenic factors affecting the *Diatraea* spp. Under the section: Factors affecting *Diatraea* spp. Richness. Are there other anthropogenic factors aside from the primary production (PP) and vegetation index e.g. does factors such as pest control affect them?

Pest control tactics have the potential to affect directly insect populations. However, no detailed information is available on control programs in the areas under study, being the case that in some of those no control is executed, so we did no considered this factor and rather focus on making a first distribution approach in relation to environmental, topographical and agricultural factors.

5. Under section distribution maps: Change this word ‘affection’ to ‘impaction’

Done

6. From section 3.4 authors should check the figure numberings and correct them.

Done

**Other comments**

Authors should include lines numbering in manuscript the next times

Done

There are grammatical and typographical errors in the manuscript. Authors should check and correct for these.

We hired a professional scientific editing service

PDF Comments

**Abstract**

1. Does not need to say this here in the abstract

Done

**Introduction**

2. Is true it effects on just these areas??

Lines 90-93 “To understand how climate change can affect sugarcane borers distribution, predictive inter- and intra-annual scale models were applied at the global, national and local scales, aiming to develop a framework to guide pest management decisions, thus reducing levels of uncertainty in the face of different climate scenarios. “

**Materials and methods**

3. you need to sort a new section regarding the study genus and introduce it including the number of species...

Lines 98 – 106 “whereas in the departments of Risaralda and Valle del Cauca, samples were collected from 2010 to 2017 from different municipalities in what it is known as the Cauca River Valley (CRV), a region that extends from northern Cauca to southern Risaralda (Fig 1). Larvae were collected in each sugarcane plot by a uniform 2 man-hour sampling effort, following the linear distribution of crop rows. Larvae were then taken to the laboratory and identified following taxonomic keys [17]. In addition, this information was used in combination with the CABI - Invasive Species Compendium (www.cabi.org) – to increase data on distribution of Diatraea spp. and increasing modeling accuracy”.

4. you need to explain how measure these and their source?

Lines 116-131 “/). At the same time, open-access weather portals were consulted such as WorldClim, from which 19 bioclimatic variables were collected on monthly rainfall and temperature [18]. The variance inflation factor (VIF) was calculated to analyze correlations between variables [19,20], providing an index that measures the extent to which the variance (the square of the estimated standard deviation) increases due to the collinearity, which allows for discarding climatic variables that could generate noise through redundancy.

Following the VIF analysis, only 6 of the 19 variables provided by WorldClim were used, plus slope (topographic slope in degrees), slope-aspect (which is defined as the compass direction to which a slope faces measured in degrees), altitude (meters), vegetation index (VI), terrestrial primary production (TPP, gCm^2^ d^1^), and TH24 (topographic heterogeneity calculated for the 24 surrounding cells) [21,22], and used along with those supplied within the ModestR® software

5. change the title of figure 3.

Done

6. seems you explained before these abbreviations in the table 1. Do not need to repeat again.

Done

7. please say more details in Methods for this approach, polar coordinates

To find that, we use the following ecuation

| $x_{n+1}=x_{n}+sin(angle x distance)$ | $y_{n+1}=y_{n}+cos(angle x distance)$ |
| --- | --- |

8. where? which country?

Colombia

**Conclusions**

9. does not make sense when you did not compare different softwares! you applied just this, so delete this part.

Done
